# Seeking Web-Based Information About Attention Deficit Hyperactivity Disorder: Where, What, and When

**DOI:** 10.2196/jmir.6579

**Published:** 2017-04-21

**Authors:** Sara Rosenblum, Elad Yom-Tov

**Affiliations:** ^1^ Laboratory of Complex Human Activity and Participation (CHAP) Department of Occupational Therapy University of Haifa Haifa Israel; ^2^ Microsoft Research Herzliya Israel

**Keywords:** attention deficit hyperactivity disorder, Internet, search engine, coping behavior, parents

## Abstract

**Background:**

Attention Deficit Hyperactivity Disorder (ADHD) is a common neurodevelopmental disorder, prevalent among 2-10% of the population.

**Objective:**

The objective of this study was to describe where, what, and when people search online for topics related to ADHD.

**Methods:**

Data were collected from Microsoft’s Bing search engine and from the community question and answer site, Yahoo Answers. The questions were analyzed based on keywords and using further statistical methods.

**Results:**

Our results revealed that the Internet indeed constitutes a source of information for people searching the topic of ADHD, and that they search for information mostly about ADHD symptoms. Furthermore, individuals personally affected by the disorder made 2.0 more questions about ADHD compared with others. Questions begin when children reach 2 years of age, with an average age of 5.1 years. Most of the websites searched were not specifically related to ADHD and the timing of searches as well as the query content were different among those prediagnosis compared with postdiagnosis.

**Conclusions:**

The study results shed light on the features of ADHD-related searches. Thus, they may help improve the Internet as a source of reliable information, and promote improved awareness and knowledge about ADHD as well as quality of life for populations dealing with the complex phenomena of ADHD.

## Introduction

### Background

Attention deficit hyperactivity disorder (ADHD) is a common, chronic, and pervasive pediatric neurodevelopmental psychiatric disorder that is prevalent among 2-10% of the population and has a complex etiology [1,2]. In the United States, approximately 5.4 million or 9.8% of children aged between 4 and 17 years are diagnosed with ADHD, with a 21.8% increase rate of diagnosis between 2003 and 2007 [3]. ADHD is characterized by a developmentally inappropriate performance level, impulsivity, and inability to sustain attention and concentration [4].

Previous literature has indicated the mobility of the real-world daily manifestations of ADHD from early childhood to adulthood as well as gender-related differences [5,6]. ADHD has longstanding neuropsychological impairments and therefore, parents of children suspected or diagnosed with ADHD often experience daily distress [7-9]. Having a child with ADHD has several implications on family life, and requires parental management of their child’s daily behavior. Parents often need to take on the role of case manager or “gatekeepers” and need to manage the home, school, and social behaviors, as well as medications, the daily routine, and therapy sessions, including communication with the therapist, if in treatment [3,10]. Not surprisingly, family members of children with ADHD face daily strains and search for information and solutions to these issues [11]. Thus, it is of interest to identify what, where, and when information is searched for related to ADHD and its management.

ADHD symptoms often appear in early childhood, yet the clinical guidelines of the American Academy of Pediatrics recommend diagnosing ADHD only from the age of 4 [12]. Although parents may feel differences in their child’s behavior in comparison to other children from an early age, most children are diagnosed with ADHD only at the age of 7-8 years when their behavior can be validated by school teachers’ reports. Consequently, ADHD diagnosis is likely to be influenced by the child’s social and school environment as well as the child’s external characteristics [13-15].

Therefore, from the child’s early years of development up to school years and even during adulthood, parents worldwide may search for resources and solutions related to their child’s behavior.

A previous study of the information needs of parents of children with ADHD revealed that parents’ information resources were related to pediatricians (86%), books (76%), general practitioners (65%), schools (61%), the Internet (59%), and other forms of media (54%) [16]. Parents were mainly interested in the causes and symptoms at the time of ADHD diagnosis and preferred to receive verbal information from professionals (69%), whereas pediatricians were rated highest as a useful, trusted, easy-to-understand, and up-to-date information source.

Nowadays, the Internet is a popular source of information and medical information is one of the most popular Internet topics thus enabling children’s diagnosis or self-diagnosis as well as support at different coping stages [17-20]. However, due to the limitations of the information supplied online, it was found that online search complements rather than opposes the primary role of the doctor-patient relationship for delivering health and medical information from a professional trusted expert [16,21-23]. The Internet may address people’s sense of self-responsibility and their “everyday” needs for health information searches through experience in navigating a multiplicity of Web-based sources [21].

In that context, it was found that parents of children with psychiatric diagnoses respond favorably to the Internet as a source of information [24]. More specifically, parents of children with ADHD found it to be a preferred source of information among other resources, including physicians or educators [16,25]. Accordingly, the extent of parental use of the Internet and the type of information sought after is of interest to health care providers [18].

### Aim of the Study

The aim of this study was to explore the dynamic process of Web-based information searches related to ADHD over a 3-month period. The specific goals were to identify *where*, *what*, and *when* information related to ADHD is sought after. Improving insight about people’s information-seeking behavior may have implications on improving health care literacy and health outcomes through Web-based solutions for this population.

## Methods

### Data Collection

Data were collected from two separate sources: (1) queries made in the United States using the Microsoft Bing search engine (following findings [26]) and (2) questions posted on the community question-answering website, Yahoo Answers. The latter site was chosen both for its overall popularity, as well as its popularity specifically among parents of children with suspected or diagnosed ADHD (eg, [27]). The study was approved by the ethics committee of the University of Haifa, Faculty of Social and Health Sciences, approval number 368/15.

**Figure 1:**
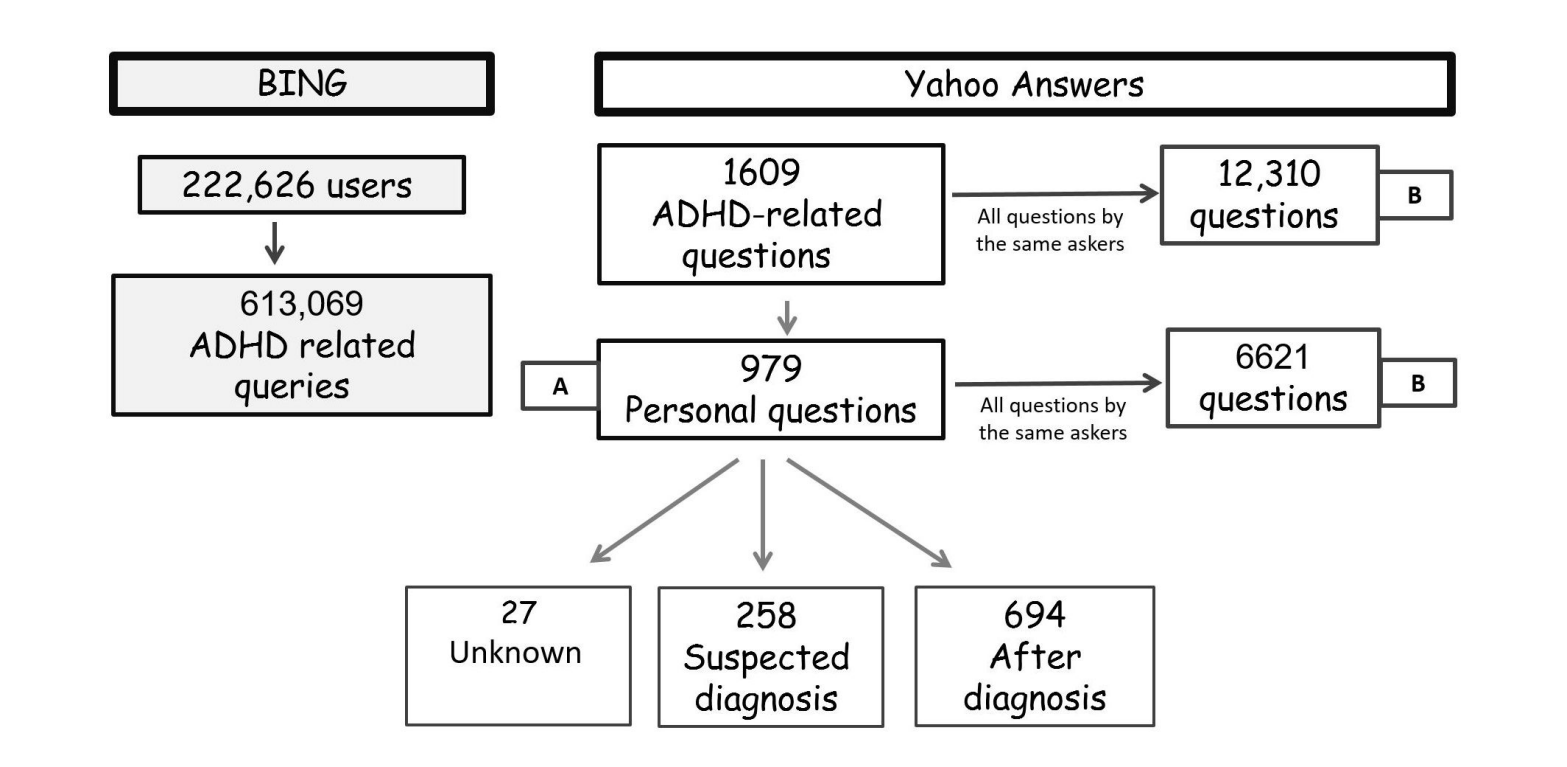
Analysis phases and number of questions analyzed by data source. ADHD: attention deficit hyperactivity disorder.

Figure 1 represents the information extraction process.

### Bing Queries

All English language queries submitted to the Bing search engine by users of desktop computers in the United States for the 3-month period between January 2015 and March 2015 were extracted. Queries were filtered to maintain only those that contained the terms “ADHD” or “attention deficit” and “disorder.” A total of 613,069 queries from 222,626 users were identified.

For each query, the text of the query (without use of any automated spelling correction), time, and date were extracted, in addition to a list of pages shown to the user and the pages clicked on by the user. In order to maintain privacy, user identifiers, which were based on browser cookies, were anonymized before access by the investigators. Queries were aggregated preceding analysis preventing individual-level user datum from being examined by the researchers.

We note that the market share of Bing in the United States is around 19%, according to recent estimates [28]. The correlation between the number of Bing users per county in the United States and the number of people in that county according to the 2010 US Census is *R*^2^=.83 (*P*=.001). Furthermore, an analysis of behavioral data collected from an opt-in consumer panel recruited by an Internet analytics company comScore, which includes age (in 5-year increments) and gender, shows a correlation of .79 (*P*=.004) between the fraction of users at each age and gender between Bing and Google users. Thus, it is estimated that Bing users are a representative sample of the US population.

### Yahoo Answers Questions

Questions about ADHD posted on Yahoo Answers, an online community question and answer website, were collected. Bing was used to query questions from Yahoo Answers that contained the term “attention deficit hyperactivity disorder” or the term “ADHD.” This resulted in 1609 unique questions.

The questions extracted from Yahoo were then each labeled by at least three human assessors from the crowdsourcing platform CrowdFlower, who were asked to label the question according to whether the question refers primarily to a person who (1) was diagnosed with ADHD, (2) is suspected of having ADHD, and (3) none of the above.

As presented in Figure 1, we refer to questions with the first label as “diagnosed,” the second as “suspected diagnosis,” and the third as “unknown.” The assessors agreed on a majority of the questions’ labels (79%) of the questions.

The authors examined a sample of the questions and identified four main categories. Two MSc in occupational therapy research assistants further classified the questions into one of the following four groups:

1. Personal account
2. General question about ADHD
3. Question about ADHD among their pets
4. Other

A question was defined as “personal” if it was asked in the first person form and included words such as “I” or “my son” or “daughter” or “we” or “my wife,” which reflects the asker’s personal involvement within the question being asked.

#### Phase A

A total of 979 personal account type of questions were found and analyzed by the two research assistant raters. Each question was examined to detect any additional information about the person discussed in the question, including the age and gender, whether the person was diagnosed with ADHD or not. Furthermore, the raters analyzed whether the question addressed problems in (1) daily function, related to activities that children do throughout the day, including activities of daily living (such as self-hygiene, dressing, showering, eating, walking), social activities, and play; (2) behavioral manifestations, related to how the child interacts with people in his or her environment at home, school, or at social events; and finally, (3) academic performance, related to issues pertaining to school or spontaneous learning, based on predetermined keywords and examples (see Multimedia Appendix 1). The keywords and examples were compiled by the first author based on initial examination and content analysis of the extracted questions. In this process, the main area of the question was recognized and keywords identified. Additionally, an extensive literature review of these areas and keywords was performed and accordingly, the guidelines for classification by the raters were established. In case of disagreement between the two raters, the issues were discussed with the first author and the classification was made according to the final decision of this discussion.

#### Phase B

All other ADHD-related questions that were posted by anyone in the *personal account* or the *general questions* participant’s category were extracted, resulting in 12,310 questions from 1130 users.

## Results

### Bing Queries

Bing queries were analyzed while focusing on the *what* and *where* queries. Namely, *what* is the main concept that people use for the search and *where* or in which sites do they search for answers to their inquiries. Initially, the 613,069 queries made on Bing were classified into whether they referred to individual persons if they contained one of the following phrases: “my son,” “my daughter,” “my child,” or “my kid;” approximately 0.4% of the queries contained these phrases, and thus are referred to henceforth as “Individual Persons Query” (IPQ). People, who made at least one IPQ made an average of 5.4 queries, compared with an average of 2.7 queries among people who did not make an IPQ. Accordingly, users who referred to individual persons tended to search for significantly more information about ADHD. The 10 most popular ADHD-related queries submitted to Bing that included specific search terms are presented in Table 1. Of these 10 queries, those referring to similar content were grouped together resulting in 6 query categories as described in Table 1. As a whole, the 10 queries detailed in Table 1 were used by 21.80% of the searchers. Within the queries, the most frequently used term in the question was ADHD (5.71%), whereas 61.5% related to the general queries about (1) ADHD and (2) ADHD and symptoms categories.

**Table 1:** The 10 most popular queries submitted to Bing.

Query category	Query	Total N=613,069, n (%)
General	ADHD^a^	35,010 (5.71)
	Attention deficit disorder	4616 (0.75)
	What is ADHD?	2960 (0.48)
Symptoms	ADHD symptoms	10,945 (1.79)
	Attention deficit disorder symptoms	2891 (0.47)
Test	ADHD test	2321 (0.37)
Medications	ADHD medications	6228 (6.9)
Children	ADHD in children	4051 (0.66)
Adults	Adult ADHD	3255 (0.53)
	ADHD in adults	3007 (0.49)

^a^ADHD: attention deficit hyperactivity disorder.

The 20 most popular websites displayed as a result of the ADHD queries, and their rank in website popularity displayed to the users as a result of their search in the Bing search result engine, are presented in Table 2. Also presented is the popularity ranking of the websites in terms of the likelihood of the users to click on these websites when they are displayed in search results (Table 2). Thus, for example, the site www.cdc.gov is the 6^th^ most popular site displayed in response to an ADHD-related query, but is not among the 20 sites most clicked in response to ADHD-related queries. Both general information websites and those specializing in ADHD are present in both rankings.

**Table 2:** Most popular websites displayed and clicked on in Bing attention deficit hyperactivity disorder (ADHD) queries.

Website	Rank in display	Rank in click	ADHD^a^-specific	HoN^b^ approved
webmd.com	1	1	No	Yes
additudemag.com	2	2	Yes	
psychcentral.com	3	3	No	Yes
wikipedia.org	4	4	No	
helpguide.org	5	7	No	
cdc.gov	6	>20	No	
nimh.nih.gov	7	15	No	
mayoclinic.org	8	10	No	
medicinenet.com	9	>20	No	Yes
healthline.com	10	8	No	Yes
chadd.org	11	18	Yes	
add.org	12	13	Yes	
add.about.com	13	6	Yes	Yes
drugs.com	14	16	No	Yes
kidshealth.org	15	20	No	Yes
ncbi.nlm.nih.gov	16	9	No	Yes
livestrong.com	17	11	No	
answers.yahoo.com	18	12	No	
healthcentral.com	19	17	No	Yes
help4adhd.org	20	19	Yes	

^a^ADHD: attention deficit hyperactivity disorder.

^b^HoN: Health on the Net.

The Health on the Net (HoN) foundation is an organization that certifies websites that provide health-related information and certification if they meet specific reliability standards. Interestingly, although 45% of the 20 popular websites that appear in Table 2 are HoN-certified, only 35% of the clicked websites are certified.

As presented in Table 2, Yahoo Answers is the 18^th^ most popular website displayed to users in response to ADHD queries on Bing and the 12th most popular site clicked by them. However, Yahoo Answers is also the only question-answering site on the list.

People who made at least one IPQ on Bing also differed in the association of their browsing behavior with Yahoo Answers. The average number of Bing queries made by people in the IPQ population who had a Yahoo Answers result displayed to them was 5.7, compared with 5.4 in the remaining population. However, people in the IPQ population who clicked on a Yahoo Answers result made an average of 14.5 queries on Bing compared with 7.2 made by other users in this sample. Thus, people who have a strong personal interest in ADHD tend to search more on Bing and click more on Yahoo Answers results. Consequently, the following sections will focus on questions from Yahoo Answers.

### Yahoo Answers Data

Analysis of the questions in Yahoo answers focused on *when* people ask questions concerning *what* trouble them, and whether the main issue that troubles them is associated with the need to ask more questions.

#### Phase A

**Figure 2:**
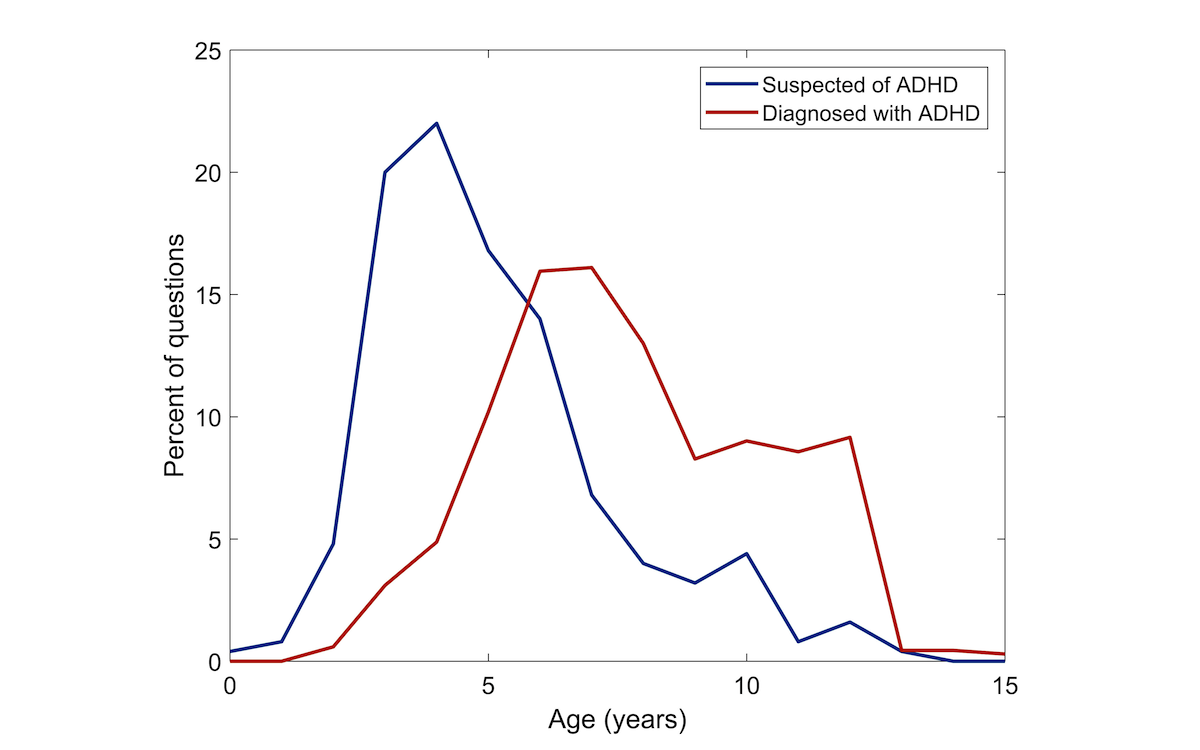
Distributions of children’s age for the 952 questions where askers describe whether their child is suspected of attention deficit hyperactivity disorder (ADHD) or diagnosed with it, in Yahoo Answers questions.

In total, 979 personal questions were found in Yahoo Answers in Phase A, as presented in Figure 1. Of these 979 questions, 26.35% (258/979) reported that the child was suspected of having an ADHD diagnosis, 70.89% (694/979) reported that the child was diagnosed, and the remaining did not report this information. From the entire sample, 69.97% (685/979) of questions were about males, whereas only 23.49% (230/979) of questions were about females, and the remaining 6.54% (64/979) of questions did not state the child’s gender. The age of children was given in 98.06% (960/979) of questions. The distribution of the reported age of children, stratified by whether they were reported as being of suspected diagnosis or diagnosed is presented in Figure 2.

Moreover, children who were diagnosed with ADHD were significantly older than those suspected of having ADHD. The average age for the latter was 5.1 years (range 0.5-20, SD 2.5), compared with 7.9 years for diagnosed children (range 3-20, SD 2.7; ranksum, *P*<.001).

**Figure 3:**
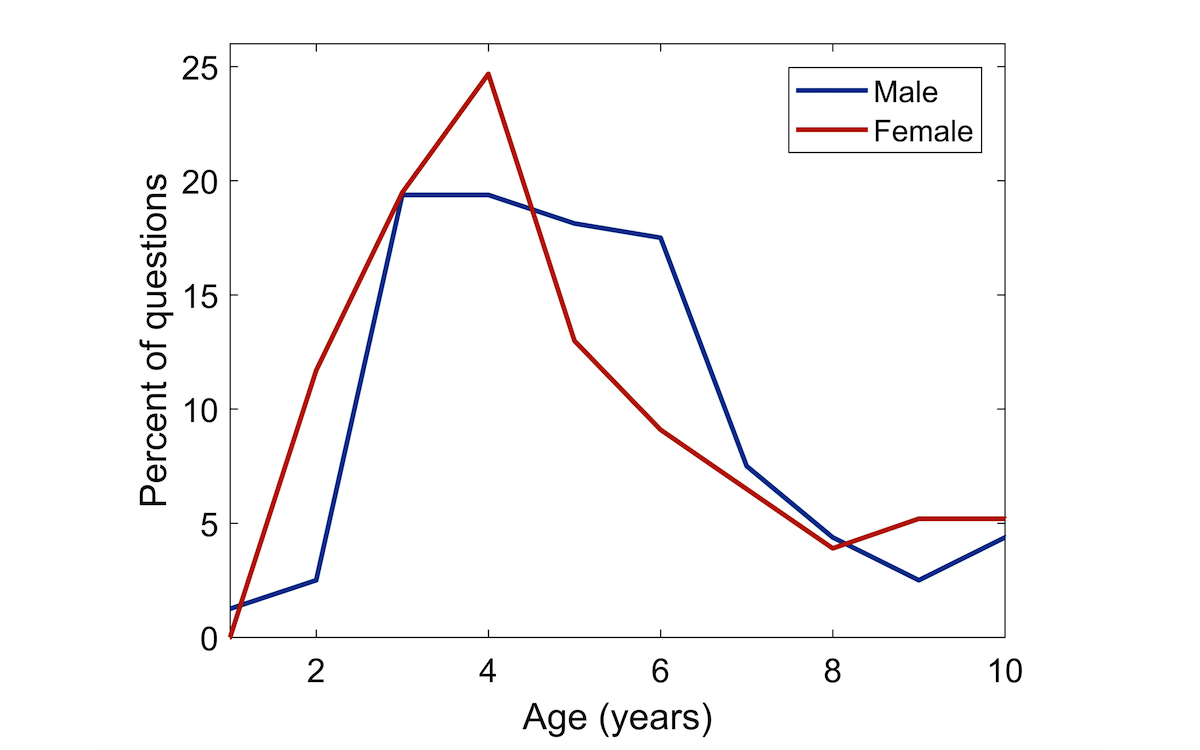
Age of children suspected of having attention deficit hyperactivity disorder (ADHD), stratified by gender.

**Figure 4:**
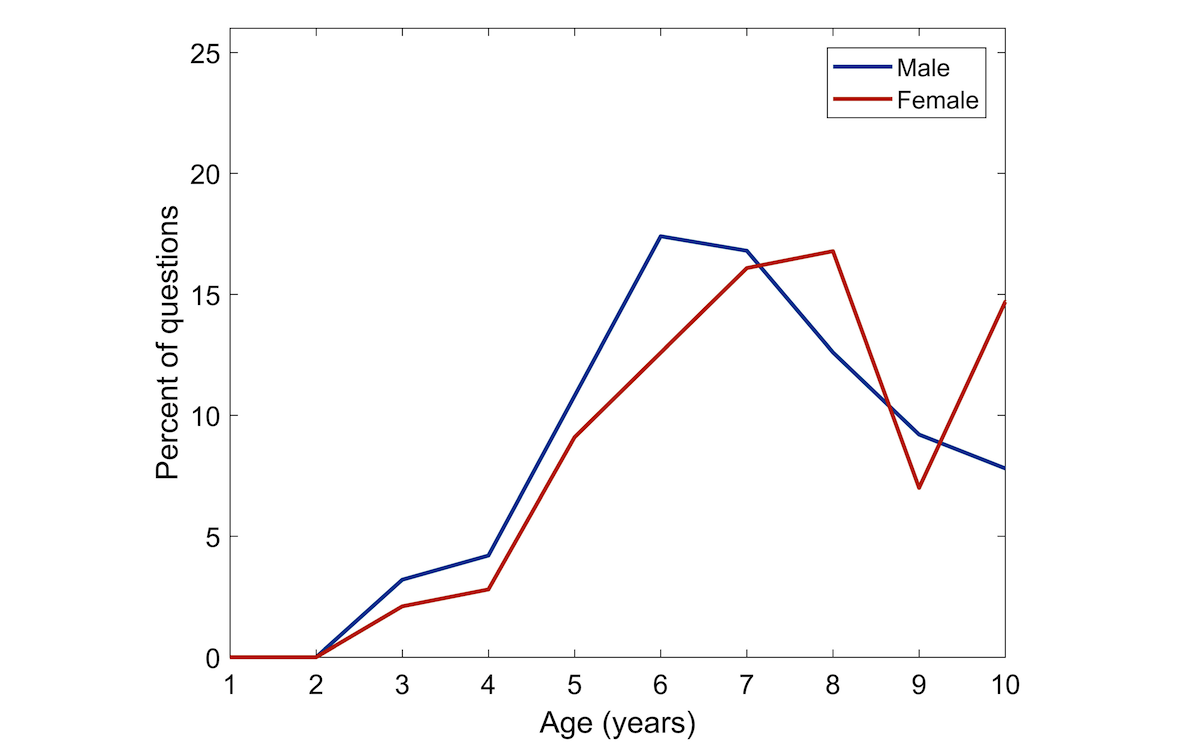
Age of children diagnosed with attention deficit hyperactivity disorder (ADHD), stratified by gender.

Figure 3 displays the age distribution of children suspected of ADHD, whereas Figure 4 displays the age distribution of children diagnosed with ADHD, both stratified by reported gender. The average age is 5.1 years for males, who are suspected of having ADHD, and 5.2 years for females (not statistically significant, ranksum test).

**Figure 5:**
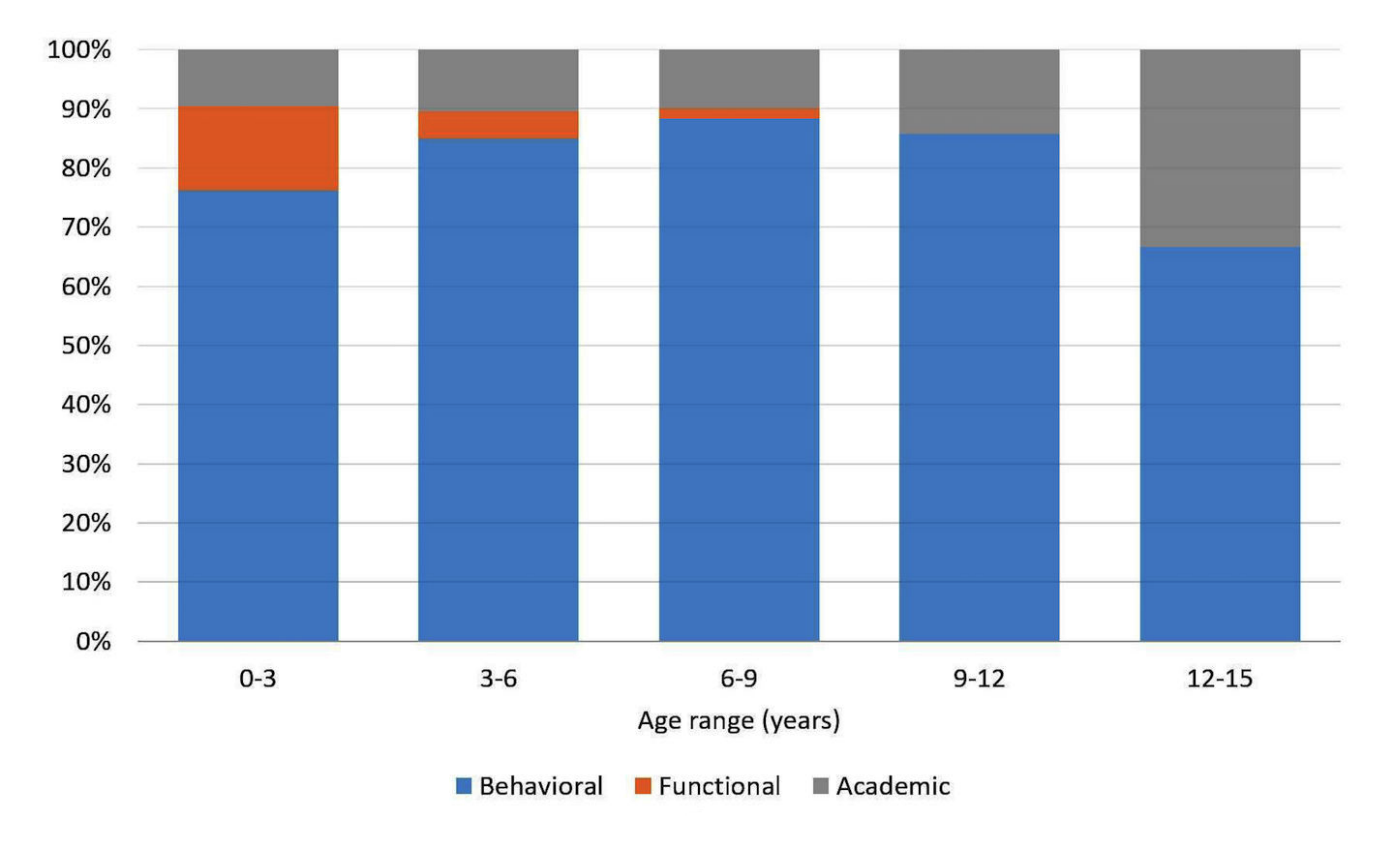
Frequency of issues reported in children suspected of attention deficit hyperactivity disorder (ADHD), stratified by age.

**Figure 6:**
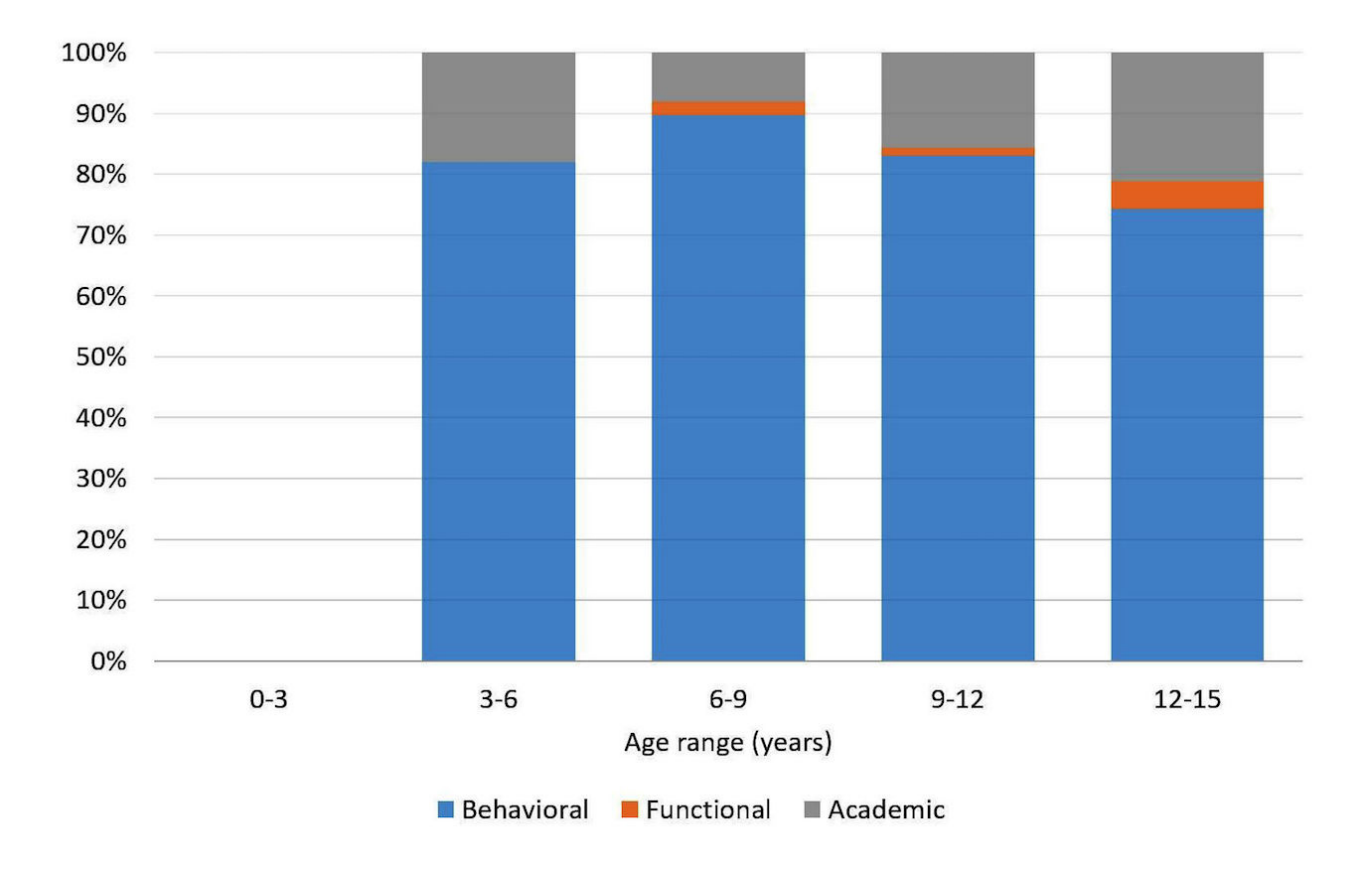
Frequency of issues reported in children diagnosed as attention deficit hyperactivity disorder (ADHD), stratified by age.

The relative frequency of each of the three types of issues reported in the questions (daily function, behavioral, academic), stratified by age is presented in Figure 5 concerning children suspected for ADHD and in Figure 6 concerning children diagnosed as ADHD.

In order to gather more information about the query process, a Cox hazard regression model [29] was constructed to assess the correlation between variables of the original first question and the likelihood of asking additional questions related to ADHD. The variables in the model included the following:

1. demographics: age and gender
2. whether the question discussed functional, behavioral, or academic issues related to ADHD
3. diagnosis status: whether the question discussed a child who was suspected to have ADHD, or already diagnosed with ADHD
4. whether the person inquiring appeared to be emotional, helpless, or positive
5. whether the person inquiring asked for information
6. whether the question mentioned that the child was receiving medication and whether the child had additional diagnoses

For this analysis, only 97.2% (952/979) of questions in which the diagnosis status was known were considered. The statistically significant variables (*P*<.05) were the diagnosis status, meaning whether a query appeared in children suspected of ADHD or after diagnosis (hazard ratio: 0.60, *P*=.01), and whether the asker revealed helplessness (hazard ratio: 1.45, *P*=.05). Thus, additional ADHD-related questions are correlated with the child being before diagnosis (hazard lower than 1) and with the person inquiring feeling helpless (eg, a hazard ratio higher than 1).

#### Phase B

In Phase B, 13.07% (1609/12,310) of ADHD-related questions by users in our sample were extracted from all 12,310 Yahoo Answers questions. The question was labeled as ADHD-related if the text contained the word ADHD, the name of a drug used to treat ADHD (adderall, amphetamine, buspirone, clonidine, ethylphenidate, melatonin, methamphetamine, methylphenidate, oxycodone, or rember), or common comorbidities of ADHD (developmental deficit malnutrition, autism, learning disability, dyslexia, bipolar, autistic spectrum disorder, mood disorder, Tourette, Asperger’s syndrome, speech impairment, or obsessive compulsive disorder). Of the entire set of questions, 53.79% (6621/12,310) of questions were asked by people in the labeled set of 979 personal account questions. Among the 979 personal questions, there were 13.0% (127/979) of questions labeled as ADHD-related in this set by people who asked a question about a child prediagnosis, and 33.6% (329/979) questions about children postdiagnosis.

**Figure 7:**
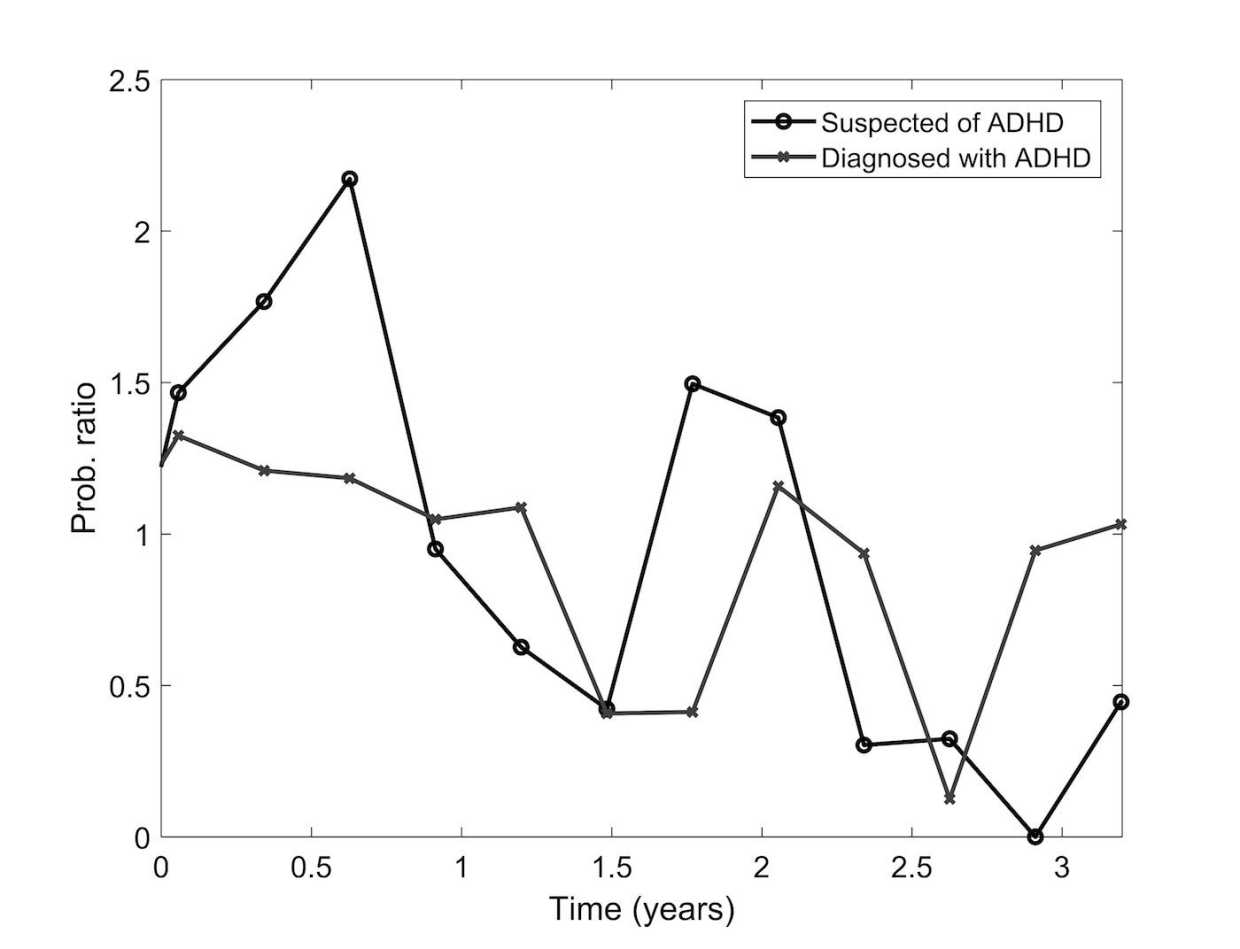
The probability ratio of asking an additional attention deficit hyperactivity disorder (ADHD)–related question, divided by suspected of ADHD or already diagnosed.

Considering all the 6621 questions, the ratio between the probability of asking an ADHD-related question, divided by the probability of asking any type of question, as a function of time is presented in Figure 7.

The questions were stratified by whether they were asked by a user who discussed a child prediagnosis in the labeled set, or a child postdiagnosis. As presented in Figure 7, a different trend is observed among people who asked about a child before diagnosis, where two spikes represent questions after approximately 7 months and after approximately 2 years. We attribute these peaks to further stages in the care for the child, for which parents require additional information. The postdiagnosis curve not only shows a downward trend (*R*^2^=.30, *P*=.005), indicating that people are less likely to ask an ADHD-related question after diagnosis, but also shows a peak around approximately 2 years, as for the undiagnosed situation.

## Discussion

### Principal Findings

The aim of this study was to obtain better insights about *where*, *what*, and *when* people seek Web-based ADHD-related information. The results indicate that the Internet indeed constitutes a source of information, as reflected by 613,069 queries about ADHD by 222,626 Bing users and 1609 questions in Yahoo Answers in the defined time of the study. These results are supported by prior studies, which suggest that the Internet is a source of information for people concerned with mental health and developmental disabilities. People, including 96% of parents, tend to seek out information on incidence, recurrence, or increased severity of the disability [26,30,31]. More specifically, both parents of adolescents with ADHD and the adolescents themselves have expressed similar strong preferences for ADHD information sources from the Internet (49% and 51%, respectively) and from a doctor (40% and 27%, respectively) [25]. Parents looking for information about their child’s symptoms and behavior seek to obtain tools for managing the condition, in light of the lack of information on the various aspects of handling ADHD [3,32].

This study also found that people, who are interested in ADHD due to a close family member’s diagnosis as reflected in IPQ queries, perform a more extensive search process compared with others. This is in line with search characteristics for other medical conditions [33]. Specifically, the study results emphasize the necessity for more knowledge in order to cope with the existing misconceptions concerning the treatment of children with ADHD and assist families who need to cope with dilemmas, confusion, and uncertainty [3,34-37].

Currently, the literature about Web-based information seeking among parents of children with ADHD is scarce. To the best of our knowledge, there is no knowledge in the existing literature related to which specific information parents seek for. Results of this study that are presented in Table 1 indicate that among the 10 most frequent queries, amounting in over 12% of the queries, 61.5% were interested in ADHD or ADHD symptoms. These are in line with previous findings regarding the uncertainty of parents regarding the nature of ADHD [38]. Providing parents with more accurate and reliable information about early symptoms of ADHD is important. These early symptoms stand out as the most important risk factor for later antisocial development and impaired daily functioning as well as learning difficulties [39,40].

The next highest category (6.9%) of queries was related to medication. Nowadays, medication use is highly common worldwide and is still the mainstay of ADHD treatment in the United States [3]. Consequently, this finding supports this population’s need for more research and knowledge about addressing dilemmas bound with taking medication, which is a source of daily stress [41-43]. Furthermore, as previously revealed, 37 of 57 ADHD websites were in fact funded by drug companies. Hence, it is not clear whether the information provided on the Web indeed addresses this population’s concerns and needs or commercial interests [44].

Aside from searching for information about ADHD symptoms and medication, 4.5% of the queries asked were related to children and 6.9% related to adults with ADHD. This finding is in accordance with the phenomena of ADHD as a life-span longitudinal disorder, affecting 2-4% of adults [45-47].

The study results provided not only a response to the question of *what* information was sought after, but also *where* the search was done. The list of the most popular websites that appears in Table 2 indicates that only 25% of the sites are ADHD-specific websites, whereas the remaining websites deal with other issues. The fact that only 35% of the clicked websites have been authorized by HoN indicates a preference of users for less authoritative websites, though possibly ones which provide more social support [48,49] or websites that serve the users’ prior beliefs. As a result of this pattern, research has found that both parents and experts agree that the quality of the Web-based information regarding treatment choices for ADHD is generally poor and does not address parents’ needs [27,48,50].

In the personal questions category on Yahoo Answers, where data about gender were available, the gender ratio was 3:1. Respectively, after addressing the questions of *what* information and *where* they searched for the information, the current results addressed the question of about *whom* and *when* on the time axis the question was asked. In line with previous findings that reported higher frequency of ADHD in males compared with females, with a gender ratio of 3:1 and 2.3:1, as well as greater impairment among boys [51,52], most questions in this study indeed were related to boys. Concerning the question of *when*, analysis revealed that questions by parents who suspected that their child had ADHD were asked already at the age of 2 years, whereas the average age in all those 258 questions is 5.1 years. This means that parental concerns about their child’s behavior and daily function in the preschool years begin well before the child enters the educational system, although children with ADHD are usually diagnosed during school years at just over the age of 7. As retrospective reporting of earlier symptoms is difficult for parents, the Web may be a useful tool for capturing information, which can support and establish the validity of the criteria of previous manifestations for future diagnosis [53].

Moreover, it was interesting to obtain information not only about *which, where*, and *when*, but also about *what*? What indeed troubles the parents? What information is sought after? The *what* question was analyzed among those who declared having a diagnosis and those who did not declared so. As presented in Figures 4 and 5, the most frequent issue raised in both of those groups was the behavioral aspect of ADHD. However, it is interesting to note that although 20% of the questions asked among families whose child’s diagnosis is unknown concerned functional daily issues, in the already diagnosed group, this category did not appear in relation to younger children. Nonetheless, there is evidence in the literature that functional deficits occur among this population over the years in the areas of self-hygiene, morning and evening organization, and play and social relationships [54-57]. It is unclear as to why these issues do not reflect in the Web-based searches. It may be that the parents do not expect to find an appropriate solution to such problems or that they feel that these issues are trivial and as parents, they are expected to cope with them regardless to the ADHD diagnosis.

Another interesting finding is that in both the group that declared and the group that did not declare a diagnosis, 20% or more of the questions around the age of 12 years were related to the academic queries category.

Academic deficits among children with ADHD have long been documented (eg, [58-60]). As academic requirements increase over the years, it is not surprising that this issue troubles parents and that they search the Web for information about how to deal with these difficulties [58]. However, it is surprising that more questions related to academic performance were asked in the group who did not declare a diagnosis. Perhaps these questions are part of the parent’s dilemma as to whether the symptoms they see are indeed indicators of ADHD, whereas among those who are already diagnosed, questions about academic performance are possibly discussed with educational and therapeutic professionals or on specific ADHD parents’ blogs or forums.

Further to achieving insight about the *which*, *when*, *where*, and *what*, our purpose was to identify who are the people that ask additional questions following their first question. The results showed that a question asked among those with no declared diagnosis and included signs of helplessness, is associated with asking more questions online. As mentioned above, parents’ daily confrontations with the disorder serves as the motivation for Web-based use. Therefore, it is understandable that parents, who are more worried and more stressed as reflected in their first question, continue to search for additional assistance.

When analyzing the phenomena of whether people whose children were diagnosed asked more questions, it was found that these people are less likely to continue to ask questions online. Perhaps this tendency occurs because at this stage, parents refer their questions to their doctor in light of the findings of preference of this source among this population [25]. Another explanation may be that parents who have a question concerning ADHD may find it easier to ask other people for solutions, rather than seek for answers in preprepared information pages. However, the peak in amount of questions asked in both groups that appeared after approximately 2 years may indicate that as the school environment requirements increase, parents again try to search for information regarding this demanding phase in life.

### Summary

In summary, our results indicate that the Internet is indeed a source of information about ADHD and tracking the search process performed by the users on the Web sheds light on people’s needs. Besides the high priority given by parents of adolescents with ADHD to receiving information from the doctor, regardless the time constrains [25], they indeed use further Web-based searching [3,25,32].

Thus, as ADHD is a chronic long-life phenomenon, expert professions may use the Web as a useful source of information for supplying accurate information over time according to this population’s daily needs [25]. Acquiring information may prevent emotional consequences and social difficulties which in turn burden costs for individuals, health services, and governments (eg, [9]). Negative life outcomes and underachievement may be prevented by understanding the specific kind of assistance is required and by providing the help in a timely manner [9].

Despite the interesting results, this study has several limitations. As far as known, this is the first study in which a quantitative analysis of the search extent and content was performed. The classification criteria as well as interrater validity need to further be discussed and improved.

Further studies that include more accurate criteria as well as a qualitative analysis of the queries made within international Internet forums are required to achieve better insight about this population’s needs and the existing barriers to seeking help (eg, [17,61]).

## Appendix

Multimedia Appendix 1 Examples to words that appear in each category.

## Abbreviations

ADHD: attention deficit hyperactivity disorder
HoN: Health on the Net
IPQ: Individual Persons Query
