# Supplementary material for: Seeking Web-Based Information About Attention Deficit Hyperactivity Disorder: Where, What, and When
Source: J Med Internet Res. 2017 Apr 21;19(4):e126. doi: 10.2196/jmir.6579 (PMC5420068; doi:10.2196/jmir.6579)
Supplement: Multimedia Appendix 1 [file jmir_v19i4e126_app1.pdf]

Multimedia Appendix 1

Examples to words that appear in each category	
**Categories**	**Example** (Note: text from the web was copied without editing)
Daily Functional behavior	Running around, speech delay, full of energy, sports and strenuous activities are out for the time because when he was riding his bike last week he got too close to the car and cut his foot on the license plate, acting differently compare to children his age , he hardly talks, handful of time i have been able to get him to sit through a story reading, she literally bounces the walls in the house, sometime I wonder if she ever get tires but she just won't stay still, he is not a normal two year child, he doesnt leason to me all of the time, she never knows what she wants. and she is a little slow, likes to put things on fire, at most he stays focused for 10 minutes. he fights sleep, first of all he runs around like crazy, all the time. he's also got a habit of climbing everything. i mean people, tables, chairs, on the counters, dishwasher, stove. he will fuss over everything. he hardly eats.,
Behavioral difficulties	having problems, behavior problems, throw anything, out of control, so much trouble, getting in trouble, throwing temper tantrums, sneakily hurting our pet, making loud actions and noises, hyperactive, throw things, acts out, broke, angry, very bad, cries a lot, always want me to kip him up, won't play for lot, does not follow directions, cursing, kicking and hitting his classmate, get a letter from his teacher about his behaviors, she bites, fight, become extremely violent, hitting, kicking, punching, biting, scratching, etc. He lies. i have gotten a phone call from his teacher almost every other day saying he is behaving badly, he has these outbursts of anger that subside just as quickly,<br>The school say they will get a phycoligist in the school to "read" her behavour and get to the bottom of it , i can't keep him interested i anything more than 5 minutes. Very defiant in kindergarten, things keep getting worse, he is very aggressive but he is so active at home i can't keep him still, whenever he plays with other children he starts fights with or hits he never takes responsibility for having done anything only to blame the other person<br>often has terrible temper tantrums and is very disruptive

Academic difficulties	when it comes time to do homework they struggled a lot, need constant help and guidance, takes 2 hours to complete (should not take so), he is 4, does not know any abc's or colors, unteachable due to his inability to sit still and pay attention, he is so unfocused that I can't get him to learn new things. She has learning difficulties, i help her lots at home and she gets lots of support at school, his teachers have a hard time with him, it's a real problem for him at school because he's incapable of sitting down for longer than literally 5 minutes, always struggled in school ,just recently the teachers have been saying to me they are worries about her as she isn't making the progress.. he just don't catch on, she makes very minimal progress if any, she also have short tension span in class.
Emotional manifestations	most "activities" just cause stress on both of us, his tics are more severe when he is stressed, I do believe in beating my child but after a while it feels like child abuse, i think he sometimes enjoys the fact that i get mad,i'm afraid he's going to get hurt.
Parent's helplessness	I don’t know what to do with this behavior, please give me some advice, driving me crazy, never do my own things, what do you do (with a child with ADHD), what should I do ?, help! we are going insane, i dont what else to do, my son is getting out of hand, i dont know what to do anymore<br>i am in a situation and i really need help, i tend to attract people that need advice to do or not to do?.
A lack of knowledge	Should we make him stop? Any alternative to help with him? (no medications) , what is ADHD, any advice about ADHD??<br>if you suspect your child having ADHD, when should they be checked?<br>What are behavior problems? they say it mimics to many other things that could be going on with him, so when is a good age to start testing,<br>what made you think your child had adhd? was the doctor able to prescribe anything?<br>i don't know much about ADHD yet i have to do my research on it<br>i want to know what can i do for her to help her to read?<br>i know this is a part of ADHD so why? (Fighting with other children)<br>what would you suggest?
Positive characters/ behavior	He loves animals and all outdoor activities, he is very bright, extremely smart, loves climb, likes to jump, she is always recognized as being an above average student.
